# Exploring Game Performance in the National Basketball Association Using Player Tracking Data

**DOI:** 10.1371/journal.pone.0132894

**Published:** 2015-07-14

**Authors:** Jaime Sampaio, Tim McGarry, Julio Calleja-González, Sergio Jiménez Sáiz, Xavi Schelling i del Alcázar, Mindaugas Balciunas

**Affiliations:** 1 Research Center in Sports Sciences, Health and Human Development, CIDESD, CreativeLab Research Community, University of Trás-os-Montes e Alto Douro, Vila Real, Portugal; 2 Faculty of Kinesiology, University of New Brunswick, Fredericton, Canada; 3 Faculty of Physical Activity Sport Sciences, University of the Basque Country, Vitoria, Spain; 4 Facultad de Ciencias de la Actividad Física y el Deporte, Universidad Europea de Madrid, Madrid, Spain; 5 Complex Systems and Sport Research Group, National Institute of Physical Education of Catalonia (INEFC), Barcelona, Spain; 6 Lithuanian Sports University, Kaunas, Lithuania; Universidad de Granada, SPAIN

## Abstract

Recent player tracking technology provides new information about basketball game performance. The aim of this study was to (i) compare the game performances of all-star and non all-star basketball players from the National Basketball Association (NBA), and (ii) describe the different basketball game performance profiles based on the different game roles. Archival data were obtained from all 2013-2014 regular season games (n = 1230). The variables analyzed included the points per game, minutes played and the game actions recorded by the player tracking system. To accomplish the first aim, the performance per minute of play was analyzed using a descriptive discriminant analysis to identify which variables best predict the all-star and non all-star playing categories. The all-star players showed slower velocities in defense and performed better in elbow touches, defensive rebounds, close touches, close points and pull-up points, possibly due to optimized attention processes that are key for perceiving the required appropriate environmental information. The second aim was addressed using a k-means cluster analysis, with the aim of creating maximal different performance profile groupings. Afterwards, a descriptive discriminant analysis identified which variables best predict the different playing clusters. The results identified different playing profile of performers, particularly related to the game roles of scoring, passing, defensive and all-round game behavior. Coaching staffs may apply this information to different players, while accounting for individual differences and functional variability, to optimize practice planning and, consequently, the game performances of individuals and teams.

## Introduction

The National Basketball Association (NBA) from the United States of America is the most competitive basketball league in the world, with a competition period in regular season comprising 82 games spanning approximately 24 weeks. The coaching staff must prepare and oversee the training loads on players throughout the entirety of the competition period, a complex process that places a great amount of physiological stress on the athletes [1]. This process also requires managing the significant differences in work demands introduced by position-specific game behaviors and player status (e.g., starting vs non-starting players), as well as adjusting throughout the season to several changing unpredictable constraints such as player injuries. Thus, the ongoing planning and monitoring of practice sessions and game performance is critical for optimizing the decisions on individual training loads taken by coaching staff.

While each player responds individually to the stress of practice and competition [2], there remains a clear need to use updated sports performance models to inform starting points for player preparation. One of the most common methods of monitoring sports performance is using game-related statistics to evaluate technical and tactical behavior, as well as the efficiency of players and teams throughout the season. Research reporting these variables frequently uses data from European league games but not from the NBA. This study using NBA data serves somewhat to address this imbalance. Performance variables represent duality of the performer and the environment in order to understand how players engage with others by detecting affordances [3]. For example, the assists are likely a result of affordances to the ball carrier created by open teammates. In fact, perception-action coupling indicates that information drives movement and movement drives information available for players to pick up [4]. In this sense, game-related statistics can provide insight on both perception and action of the players. In addition, they may provide a measure of co-adaptation, in the way that players function as part of a larger system (the team) co-adapting to small but important changes in each others structure and function [5].

Basketball performance depends primarily on shooting 2-point field-goals and on securing defensive rebounds [6–8]. In close contested games, however, fouls and free-throws exhibit increased importance for determining game outcome than for lesser contested games [8, 9]. Other remaining game statistics such as offensive rebounds, turnovers, steals and assists are not reported consistently as discriminating performance variables for winning and losing. When contrasting the best and worst teams, the best performance variables for long term success are related to assists, steals and blocks, denoting the importance of passing skills and of defensive skills along outside and inside court positions [10]. Research from NBA data likewise reported winning game outcomes to be related to better offensive efficiency, specifically points scored in the third quarter, as well as the defensive variables of fouls and steals [11]. Thus, as expected, the results suggest that both offensive and defensive variables are important for winning games.

These descriptions are informative on a team-level basis, however, a need exists to undertake player-level analysis in order to better understand what performance variables most discriminate elite players from other players. In the NBA context, this aim can be accomplished by contrasting game performances from the awarded players that comprise the first, second and third NBA team (all-stars) with the performance statistics of the other players. The all-star players from these three teams are selected from a voting conducted by a panel of sportswriters and broadcasters [12]. The players receive five points for a first team vote, three points for a second team vote, and one point for a third team vote. At the end, and accounting for playing positions, the five players with the highest point totals make the first team, the next five make the second team, and the remaining five the third team.

One of the most recent advances in assessing basketball performance is player-tracking technology [13, 14]. This technology uses computer vision systems designed with algorithms capable of measuring the positions of players with a sampling rate around 25 frames per second [15]. Of course, kinematic variables such as distance, velocity or acceleration may be derived from these data, and sampling frequencies might improve in future [16]. Currently, the tracking technology is being used with data obtained from notational analysis providing combined information about sports performance; for example, by analyzing the distance covered by players when the team is attacking and when the same team is defending. Research in basketball using positional-derived variables however is limited at present to small samples of young basketball players examining physical demands [17], effects of defensive pressure on movement behavior [18], and how tactical performances are affected by activity workload [19].

These new tracking data open up possibilities that advance understanding of game performance by embracing a more holistic approach to analyzing sports behavior. For example, movement patterns (kinematics) from tracking data complement variables from the physiological (e.g., work rate), technical (e.g., actions) and tactical (e.g., individual/team behavioural patterns) domains leading to a more complete description and understanding of sports behavior in its entirety. As noted, an issue to address in this study using the large amounts of tracking data at hand concerns different basketball game performance profiles for different players and teams. That is, to categorize individual player performances into like groupings for use as baseline reference for the future development and preparation of players. The aim of the present study then is twofold: (i) to compare basketball game performances from the all-star and non all-star players, and (ii) to identify and describe the different basketball game performance profiles based on different game roles in the NBA.

Regarding the first aim, it was hypothesized that all-star players will outperform the non all-stars in game statistics. Therefore, the player performances on an actions-by-minute of play basis were compared, in aim of identifying performance variables that discriminate between the two separate groups of players. It is expected that all-star players should outperform the non all-star players in their performance statistics, particularly in scoring and passing related variables, as these important variables are thought to place higher demands on anticipatory processes [20–22]. In the second aim it was hypothesized that player performance profiles will present similarities and dissimilarities that can be used to identify different groups of players based on playing position. This aim is accomplished by using actions-per-game, in order to identify different groups of player performances, regardless of minutes of play in the games, thereby identifying those performance variables that discriminate between different player groupings.

Finally, it is important to describe the data within these performance-based groupings according to the players (all-star vs. non all-star) and playing positions. For example, some groups might have strong presence from all-star players and other groups might comprise both all-star and non all-star players from specific positions. This information can be useful when used in planning representative tasks in practice sessions, thereby fine-tuning playing behaviors in competition by using representative tasks in training [23, 24]. In fact, players are often divided in practice into smaller groups according to specific positions as well as their playing standard. Non-starting players, for example, lack the same amount of playing time as starting players, and this competitive playing deficit likely affects their responses to competition throughout the season [21, 25]. It follows that a detailed description of these different performance profiles using available objective measures would serve as an appropriate performance baseline for optimizing practice planning and, ultimately, for improving game performance.

## Methods

### Sample and variables

Archival data were obtained from open-access official NBA records for 1230 games played during the 2013–2014 regular season (available at http://stats.nba.com, these records contained both non-tracking and tracking data). A total of 30 teams played 82 games between October 29, 2013 and April 16, 2014. The gathered database had records of game performances from 548 players. The cases of player transfer between teams were counted as two different records.

The variables analyzed included the points per game, minutes played and the following game actions, as defined by the NBA and the company responsible for the player tracking process (SportsVU, Northbrook, IL, USA):

- Pull-up shots: any jump shot outside 10 feet where a player took one or more dribbles before shooting. Gathered variables include pull-up points per game (PPG) or minute (PPM), field-goal percentage (FG%) and 3-point field-goal percentage (3FG%).
- Catch and shoot: any jump shot outside of 10 feet where a player possessed the ball for two seconds or less and took no dribbles. Gathered variables include catch and shoot PPG or PPM, FG% and 3FG%.
- Close shots: any jump shot taken by a player on any touch that starts within 12 feet of the basket, excluding drives. Gathered variables include close PPG or PPM and FG%.
- Drives: any touch that starts at least 20 feet of the hoop and is dribbled within 10 feet of the hoop and excludes fast breaks. Gathered variables include drives PPG or PPM and FG%.
- Passing-variables: the total number of passes a player makes and the scoring opportunities that come from those passes, whether they lead directly to a teammate scoring a basket (assists) or free throw (free-throw assists), or if they set up an assist for another teammate (secondary assists). Gathered variables also include total assists opportunities and total points created by assists.
- Touches-variables: the number of times a player touches and possesses the ball (touches per game), where those touches occur on the court (front, close or elbow), how long the player possessed the ball (time of possession), and the number of points per touch or per half-court touch. Gathered variables also include blocks, steals and the opponent field goals made at the rim while being defended.
- Speed and distance: variables that measure the distance covered (expressed in miles) and the average speed of all movements (expressed in miles per hour) by a player while attacking or defending.
- Rebounds: the number of rebounds secured (rebounds), the times when the player was within the vicinity (3.5 feet) of a rebound (chances), the number of rebounds a player recovers compared to the number of rebounding chances available (percentage chances) as well as if the rebound was uncontested by an opponent (uncontested). These variables were gathered either for defensive and offensive rebounds.
- Free-throw percentage: the number of free-throws made divided by the number of free-throws attempted.

Video footage from the entire court was unavailable making assessment of the NBA tracking data impossible. The NBA non-tracking data (e.g., assists, steals or defensive rebounds) however was assessed for reliability as follows. Two games were selected at random and analyzed conjointly through systematic observation by two experts. The minimum Cohen’s κ value for all variables exceeded 0.91 demonstrating high inter-rater reliability [26] between the NBA non-tracking data and the two experts.

### Data analysis

Variables expressed as counts per game were divided by average minutes played. Records were screened for univariate outliers (cases outside the range Mean ± 3SD) and distribution tested, together with advised assumptions for each following inferential analysis [27]. To identify which variables best predict the player category (i.e., all-star vs. non all-star), the performance per minute of play was analyzed using a descriptive discriminant analysis. Structure coefficients greater than |0.30| were interpreted as meaningful contributors for discriminating between the two groups [27]. Validation of discriminant models was conducted using the leave-one-out method of cross-validation [28]. Also, a k-means cluster analysis was performed on the entire sample with the aim of creating and describing maximal different groups of game performance profiles. The cubic clustering criterion, together with Monte Carlo simulations, was used to identify the optimal number of clusters, thereby avoiding using subjective criteria. This statistical technique requires that all cases have no missing values in any of the variables introduced in the model; there were a total of 339 cases meeting this condition (62%). Afterwards, a descriptive discriminant analysis was performed to identify which of the variables best predicts the playing clusters.

One-way independent measures ANOVA was used to compare the variables not selected in the discriminant models (i.e., points scored per game and minutes played). Tukey post-hoc homogeneous subsets were used to describe post-hoc results. Statistical significance was set at 0.05 and calculations were performed using JMP statistics software package (release 11.0, SAS Institute, Cary, NC, USA) and SPSS software (release 22.0, SPSS Inc., Chicago, IL).

## Results

### Comparing all-star and non all-star players

The means and standard deviations from the variables according to the all-star vs. non all-star categories are presented in Table 1. The most important variables for differentiating all-star and non all-star performances per minute of play were identified using discriminant analysis. The obtained function was statistically significant (p≤0.001) with a canonical correlation of 0.59 (Λ = 0.65) and reclassification of 97.2%. The structure coefficients (SC) from the function reflected emphasis on elbow touches (SC = 0.43), defensive rebounds (SC = 0.35), close touches (SC = 0.34), close points (SC = 0.33), pull-up points (SC = 0.33) and speed in defense (SC = -0.33) (see Table 1). There were six cases misclassified (60.0% accuracy) in the all-star group and seven cases misclassified (97.8% accuracy) in the non all-star group, therefore, the obtained mathematical model shows high accuracy in classifying the players into their original groups.

**Table 1:** Means, standard deviations and structure coefficients from all-star and non all-star categories. The variables expressed as counts were divided by minutes played.

Group/Variable	All-star players	Non all-star players	Structure coefficients
	(n = 15)	(n = 324)	
Pull-up PPM	0.13±0.09	0.07±0.05	0.33
Pull-up FG%	0.36±0.08	0.34±0.09	0.08
Pull-up 3FG%	0.27±0.13	0.28±0.18	-0.01
Catch and shoot PPM	0.10±0.05	0.12±0.06	-0.09
Catch and shoot FG%	0.43±0.03	0.37±0.07	0.23
Catch and shoot 3FG%	0.36±0.14	0.35±0.11	0.04
Drives PPM	0.09±0.07	0.06±0.05	0.19
Drives FG%	0.50±0.06	0.42±0.15	0.15
Close PPM	0.07±0.07	0.03±0.04	0.33
Close FG%	0.56±0.09	0.55±0.21	0.02
Passes	1.44±0.34	1.22±0.39	0.17
Assists	0.15±0.07	0.09±0.06	0.28
Free-throw assists	0.01±0.01	0.01±0.01	0.14
Secondary assists	0.03±0.02	0.02±0.01	0.15
Assist opportunities	0.28±0.12	0.19±0.11	0.26
Points created assists	0.34±0.15	0.22±0.13	0.29
Blocks	0.02±0.02	0.01±0.01	0.10
Steals	0.03±0.01	0.03±0.01	0.09
Opponents’ FGM Rim	0.01±0,00	0.03±0.02	-0.22
Touches	2.10±0.32	1.70±0.43	0.29
Front court touches	1.58±0.41	1.28±0.45	0.20
Close touches	0.09±0.08	0.05±0.04	0.34
Elbow touches	0.13±0.09	0.06±0.05	0.43
Points per touch	0.01±0,00	0.01±0.01	-0.18
Points per half court touch	0.01±0,00	0.02±0.01	-0.17
Time of possession	0.12±0.06	0.08±0.06	0.18
Distance in offense	0.04±0,00	0.04±0,00	-0.18
Distance in defense	0.03±0,00	0.03±0,00	-0.26
Speed in offense (mph)	4.38±0.36	4.50±0.28	-0.13
Speed in defense (mph)	3.65±0.16	3.86±0.20	-0.33
Offensive rebounds	0.04±0.03	0.03±0.02	0.13
OR chances	0.08±0.05	0.06±0.04	0.08
% OR per chance	0.54±0.10	0.51±0.11	0.07
OR uncontested %	0.52±0.17	0.46±0.19	0.10
Defensive rebounds	0.16±0.07	0.11±0.04	0.35
DR Chances	0.24±0.10	0.19±0.07	0.25
% DR per chance	0.66±0.06	0.61±0.06	0.27
DR uncontested %	0.19±0.07	0.18±0.07	0.05
Free-throw %	0.79±0.09	0.71±0.17	0.11
Points per game ^a^	22.20±4.70	9.20±5.10	*
Minutes played ^a^	35.90±2.20	22.50±8.80	*

Legend: a—variables not entering the discriminant analysis model

*p ≤ 0.05.

**Fig 1:**
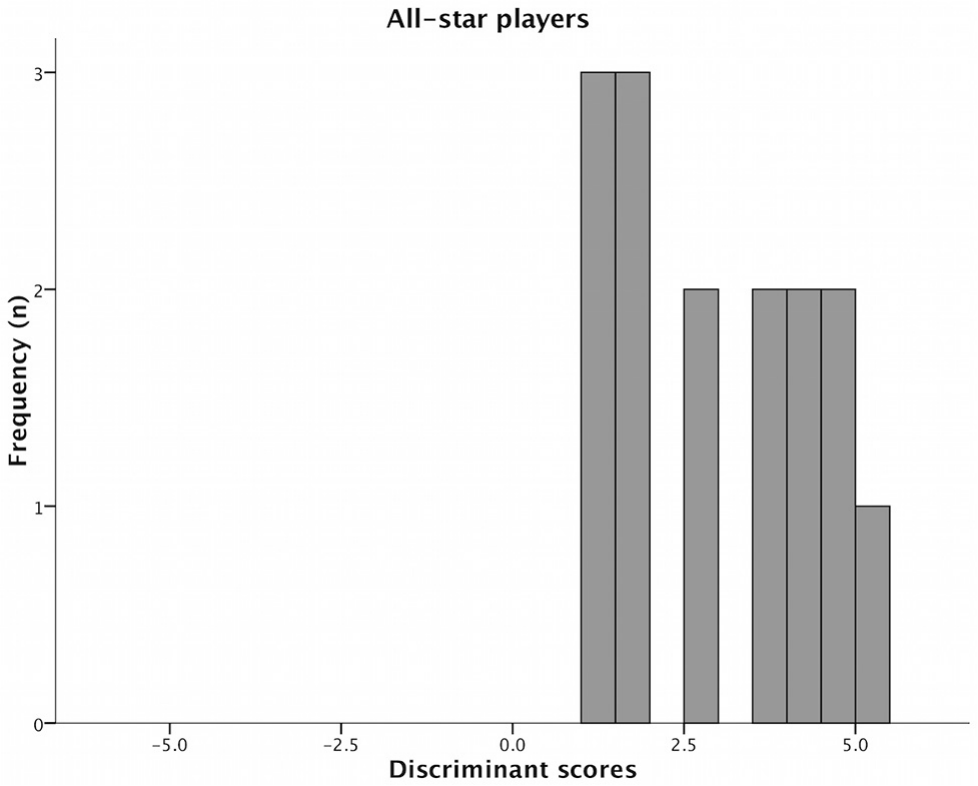
Distribution from the discriminant scores across all-star players.

**Fig 2:**
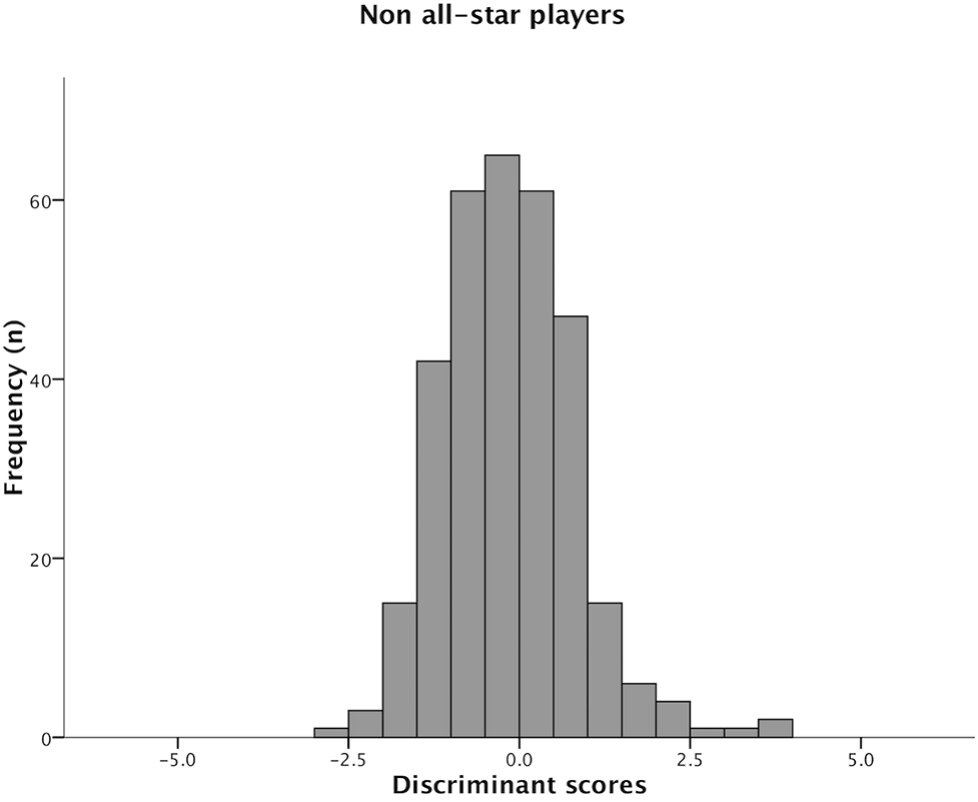
Distribution from the discriminant scores across non all-star players.

Figs 1 and 2 present the distribution from the discriminant scores in each group of players. The all-star players presented higher mean scores when compared to non all-star players (3.04±1.45 and -0.13±0.87, respectively).

### Describing different game performance profiles

The cubic clustering criterion (CCC) along with Monte Carlo simulations was used to identify the optimal number of clusters. The largest value (CCC = 252.6) was obtained for a model of seven clusters. Therefore, a k-means cluster analysis was performed to create and describe seven maximal different groups of performance profiles per game. The means and standard deviations from the variables according to the cluster solutions are presented in Table 2. The discriminant analysis revealed four statistically significant functions (p≤.001), however, the first two yielded a total of 94.7% from the total variance, with canonical correlations of 0.98 and 0.88, respectively. The reclassification of the cases in the original groups was very high (96.2%).

**Table 2:** Means, standard deviations and structure coefficients from the obtained model of clusters. The variables expressed as counts are averages per game.

Cluster/Variable	Cluster 1	Cluster 2	Cluster 3	Cluster 4	Cluster 5	Cluster 6	Cluster 7	Function 1 (78.3%)	Function 2 (16.1%)
Pull-up PPG	0.75±0.68	3.44±2.10	1.53±1.10	5.12±2.05	1.04±0.87	3.17±1.82	1.96±1.46	0.20	0.34
Pull-up FG%	0.31±0.13	0.36±0.05	0.34±0.06	0.38±0.04	0.33±0.08	0.35±0.07	0.36±0.07	0.06	0.05
Pull-up 3FG%	0.29±0.27	0.27±0.10	0.28±0.13	0.32±0.06	0.25±0.17	0.31±0.11	0.25±0.14	0.00	0.06
Catch and shoot PPG	1.60±1.14	4.18±1.79	4.32±2.07	2.99±1.15	2.49±1.61	2.60±1.42	3.12±1.43	0.13	-0.12
Catch and shoot FG%	0.35±0.10	0.41±0.04	0.40±0.05	0.39±0.05	0.37±0.06	0.38±0.07	0.39±0.05	0.07	-0.03
Catch and shoot 3FG%	0.35±0.14	0.36±0.09	0.36±0.10	0.39±0.06	0.33±0.11	0.37±0.09	0.33±0.11	0.02	0.04
Drives PPG	0.68±0.64	2.85±1.87	1.37±0.95	4.11±1.43	0.96±0.77	3.06±1.64	1.93±1.53	0.18	0.31
Drives FG%	0.37±0.23	0.49±0.07	0.43±0.07	0.46±0.05	0.44±0.13	0.44±0.06	0.45±0.10	0.06	0.03
Close PPG	0.33±0.18	1.61±1.49	1.60±0.63	0.41±0.28	0.77±0.31	0.73±0.14	1.18±0.19	0.08	-0.15
Close FG%	0.54±0.30	0.57±0.13	0.58±0.09	0.54±0.11	0.56±0.17	0.48±0.28	0.55±0.11	0.01	-0.08
Passes	13.79±5.71	46.93±8.24	29.03±6.32	61.23±6.42	22.22±6.63	47.09±11.29	31.98±7.75	0.43	0.59
Assists	0.88±0.65	4.09±1.10	1.87±0.69	6.70±1.52	1.47±0.83	5.07±2.02	2.48±1.09	0.31	0.65
Free-throw assists	0.10±0.05	0.43±0.15	0.22±0.12	0.73±0.24	0.18±0.13	0.53±0.20	0.29±0.15	0.24	0.46
Secondary assists	0.25±0.16	0.90±0.25	0.53±0.18	1.51±0.37	0.38±0.21	1.05±0.39	0.59±0.24	0.30	0.54
Assist opportunities	1.81±1.16	8.07±2.03	3.72±1.32	13.29±2.75	2.96±1.61	10.03±3.77	4.92±2.01	0.33	0.69
Points created assists	2.10±1.50	9.63±2.59	4.42±1.63	15.69±3.47	3.49±1.91	11.79±4.63	5.85±2.56	0.32	0.65
Blocks	0.15±0.04	0.49±0.32	0.58±0.53	0.22±0.15	0.31±0.28	0.29±0.10	0.39±0.34	0.10	-0.14
Steals	0.36±0.22	1.18±0.41	0.95±0.36	1.33±0.50	0.63±0.26	0.96±0.43	0.90±0.31	0.24	0.12
Opponents’ FGM Rim	0.55±0.13	0.52±0.04	0.54±0.04	0.57±0.05	0.53±0.06	0.58±0.09	0.55±0.05	0.00	0.09
Touches per game	19.43±7.56	66.93±7.94	42.86±8.56	82.36±7.49	30.78±8.35	64.06±13.17	45.28±9.69	0.50	0.60
Front court touches	14.44±6.52	50.49±6.84	31.01±6.89	71.00±7.64	22.14±7.67	52.78±12.59	33.81±9.35	0.44	0.66
Close touches	0.49±0.12	2.09±1.79	1.96±0.98	0.73±0.35	1.00±0.60	1.08±0.20	1.58±0.54	0.09	-0.12
Elbow touches	0.60±0.19	3.68±2.94	1.82±1.32	1.44±0.71	1.20±1.03	1.74±1.58	1.93±0.13	0.12	-0.04
Points per touch	0.23±0.07	0.27±0.09	0.30±0.06	0.20±0.04	0.24±0.08	0.20±0.07	0.26±0.08	0.06	-0.19
Points per half court touch	0.32±0.11	0.35±0.12	0.42±0.08	0.23±0.05	0.35±0.13	0.25±0.10	0.35±0.11	0.02	-0.24
Time of possession (min per game)	0.84±0.62	3.12±1.30	1.35±0.51	6.53±0.72	1.25±0.94	4.38±1.67	1.97±1.07	0.28	0.72
Distance in offense (season total)	14.30±7.84	97.43±10.97	87.83±8.78	94.15±12.41	42.55±9.53	33.92±13.92	67.90±7.20	0.83	-0.29
Distance in defense (season total)	12.27±6.68	81.82±8.82	77.44±7.62	75.89±10.40	37.31±8.83	28.10±11.45	59.05±7.02	0.80	-0.38
Speed in offense (mph)	4.52±0.27	4.39±0.31	4.38±0.31	4.62±0.25	4.49±0.27	4.58±0.25	4.50±0.30	-0.02	0.11
Speed in defense (mph)	3.92±0.21	3.71±0.18	3.85±0.19	3.72±0.15	3.90±0.19	3.74±0.17	3.85±0.20	-0.08	-0.11
Offensive rebounds	0.40±0.31	1.17±0.84	1.15±0.81	0.61±0.28	0.75±0.58	0.70±0.57	0.99±0.73	0.10	-0.12
OR chances	0.79±0.57	2.17±1.51	2.17±1.46	1.38±0.64	1.45±1.14	1.35±0.99	1.90±1.33	0.10	-0.11
% OR per chance	0.52±0.15	0.54±0.07	0.53±0.07	0.45±0.08	0.52±0.09	0.51±0.08	0.51±0.07	-0.01	-0.08
OR uncontested %	0.46±0.25	0.50±0.15	0.52±0.14	0.35±0.12	0.48±0.16	0.38±0.16	0.48±0.14	0.00	-0.13
Defensive rebounds	1.40±0.75	4.87±1.94	3.83±1.65	2.85±0.75	2.25±0.96	3.19±1.48	3.23±1.57	0.20	-0.08
DR Chances	2.30±1.18	7.45±2.68	6.26±2.40	4.65±1.34	3.80±1.52	4.93±2.24	5.23±2.33	0.20	-0.09
% DR per chance	0.61±0.08	0.65±0.06	0.61±0.06	0.62±0.05	0.59±0.05	0.65±0.06	0.61±0.06	0.03	0.07
DR uncontested %	0.17±0.09	0.19±0.06	0.20±0.06	0.13±0.04	0.20±0.07	0.15±0.05	0.19±0.06	0.01	-0.15
Free throw %	0.75±0.14	0.80±0.13	0.80±0.08	0.80±0.09	0.77±0.09	0.73±0.11	0.71±0.22	0.19	0.10

The structure coefficients from the functions are presented in Table 2. The first function had stronger emphasis on total distance covered in offense (SC = 0.83) and defense (SC = 0.80), whereas the second function was emphasized by performance obtained in passing-related variables (see Table 2).

Table 3 presents the differences between clusters in points scored per game, minutes played and distance from each case (player) to cluster centroid. The clusters 2 and 4 had more playing minutes and points per game. The clusters 1 and 5 were the most homogeneous, as identified in smaller distances to group centroid. In addition, player distributions in the seven clusters were contrasted against player category, presence in the NBA first team, and specific court position of players. The all-star players were grouped in clusters 2, 3 and 4. The NBA first team was grouped in clusters 2 and 4.

**Table 3:** Player distributions in the seven clusters contrasted against player category, NBA first team and player specific court position.

Cluster	Cluster 1	Cluster 2	Cluster 3	Cluster 4	Cluster 5	Cluster 6	Cluster 7	TOTAL
Points per game	4.4±2.3	17.8±6.3	12.8±3.4	16.6±3.7	7.2±2.9	12.9±4.3	11.5±4.1	hs (1)(5)(3,6,7) (2,4)
Minutes played	12.6±5.0	34.5±3.2	30.9±3.4	33.8±2.2	19.7±4.2	28.8±5.2	26.9±4.3	hs (1)(5)(6,7)(6,3)(2,4)
Distance cases to centroid	14.5±5.1	20.2±6.8	20.8±6.8	19.3±8.4	16.1±5.3	22.3±9.6	17.9±7.0	hs (1,5)(2,4,5,7)(2,3,4,6,7)
Non all-star players	93 (28.7%)	21 (6.5%)	37 (11.4%)	18 (5.6%)	74 (22.8%)	24 (7.4%)	57 (17.6%)	324 (100%)
All-star players	0 (0.0%)	8 (53.3%)	2 (13.3%)	5 (33.3%)	0 (0.0%)	0 (0.0%)	0 (0.0%)	15 (100%)
NBA first team	0 (0%)	4 (80%)	0 (0%)	1 (20%)	0 (0%)	0 (0%)	0 (0%)	5 (100%)
Guards (1–2)	33 (19.1%)	15 (8.6%)	15 (8.6%)	23 (13.3%)	35 (20.2%)	18 (10.4%)	34 (19.6%)	173 (100%)
Forwards (3–4)	28 (24.4%)	13 (11.3%)	23 (20.0%)	0 (0.0%)	31 (27.0%)	1 (0.1%)	16 (15.7%)	115 (100%)
Centers (5)	3 (23.1%)	1 (7.7%)	1 (7.7%)	0 (0.0%)	3 (23.1%)	0 (0.0%)	5 (38.5%)	13 (100%)
Unclear position or missing values	29 (67.4%)	0 (0.0%)	0 (0.0%)	0 (0.0%)	9 (20.9%)	5 (11.6%)	0 (0.0%)	43 (100%)

Legend: hs—post-hoc homogeneous subsets Tukey test.

**Fig 3:**
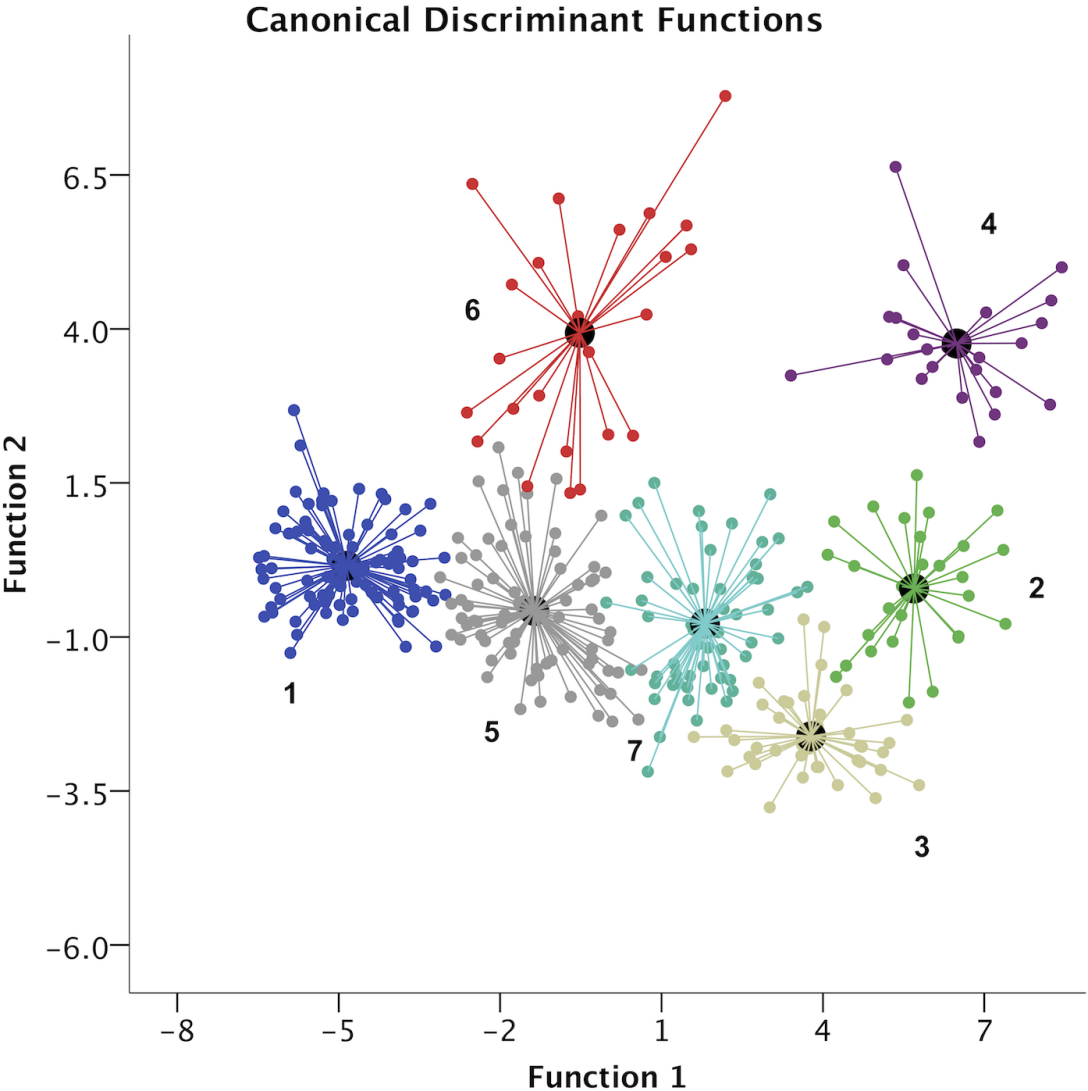
Territorial map from the cases and created clusters.

Fig 3 presents the territorial map from the cases and created clusters within the space from the first and second discriminant functions. Players from clusters 4 and 2 exhibited better overall performances, however, players from cluster 6 also performed well in variables related to function 2.

## Discussion

This study aimed to compare game performances of all-star and non all-star basketball players and to identify and describe different basketball game performance profiles in the NBA. In general terms, key performance indicators were identified that discriminate all-star players from non all-stars and, also, the different groupings of performance profiles in competition.

### Comparing all-star and non all-star players

As expected, all-star players outperformed non all-star players in performance statistics, particularly in defensive rebounds, close touches and close points, pull-up points and assists. (Note. These results may be confounded in that the distinction between all-star and non all-star players is determined by sportswriters and broadcasters. This said, discrimination between these prejudged player groups is reflected in some game performance variables as reported in this study.)

Noted previously, the variables obtained from the tracking systems allow use of court locations for better understanding several game statistics. Therefore, these results increase knowledge of basketball game behavior by identifying key performance variables and by reducing prior emphasis on the importance of distance covered and velocity. The reclassification obtained was very high and hence affirms accuracy of the mathematical model.

The close touches and points were identified as key variables, suggesting that all-star players performed consistently better than non all-star players within 12 feet of the basket. These court locations are highly concentrated with teammates and opponents with frequent physical contact between players. These complex actions require high anticipatory skills [29] and all-star players outperform non all-stars in producing these complex skills under extreme adverse conditions [20–22]. Also, related with these findings, all-star players demonstrated the ability to score pull-up points, again showing how well these players perceive environmental information and adapt their behavior accordingly [30, 31], as they strive to reach a better position from which to score (oftentimes using one or more dribble actions before shooting, for example). Several studies from basketball [32], football [33] and futsal [34] analyzing space-time dynamics of player dyads inform how the formation of playing patterns are influenced by scoring targets (i.e., baskets and goals). This higher ability to perceive the environment requires a developed attention span [35, 36], perhaps evidenced in the higher number of assists given that assists constitute passes to a teammate leading directly to a subsequent field goal.

The distance covered and average speeds were not discriminant variables between the all-star and non all-star players. Until the availability of recent technology, getting reliable time motion data in basketball games has been difficult to acquire and, as such, low accuracy in the measures reported and/or small sample sizes have been a concern since early times [37]. The present results however provide measures of distance and velocity from an entire NBA season that are considered reliable [13, 14], despite the 25 Hz sampling frequency limitation [16]. Although discriminant analysis only emphasized velocity in defense, there seems to be a tendency for all-star players to cover slightly shorter distances at lower average velocities. This might be important in that it is consistent with previous observations on the enhanced attunement of players to perceive affordances [38, 39]. Thus, all-star players may well make less mistakes when deciding when and where to run in both offense and defense, possibly taking shorter paths to reach their destinations. These fewer mistakes in a game might well result in lower distances covered by these players. In addition, these considerations might also suggest that all-star players are more efficient, having less energy demands placed on them during a game. In fact, research suggests that motor efficiency achieved through intensive training, leads to improved perception, focus, anticipation, planning and fast responses [40]. The finding of lower defensive velocities for all-star players may reinforce this observation, but might also suggest that these players might be focusing their efforts more on offensive performances, as they are more complex and depend more upon their high level expertise [22, 41].

### Describing different game performance profiles

The results reported different performance profiles for different player groupings. There were seven different groups identified by the analysis, obtaining very high reclassifications of the cases (96.2%). These groupings, based on total distance covered in the season and performance per game, might be used in developing specific playing profiles that, taking into consideration the influence of individual differences and functional variability, may serve as baseline to facilitate optimizing practice planning and game performance.

The clusters 2, 3 and 4 performed best at discriminant variables from function 1 (78.3% of total variance) and they contained all of the all-star players. These players participated in more than 30 minutes per game and scored many points per game (from 12.8±3.4 to 17.8±6.3). As an effect of these higher playing times, the most discriminant variables of this function were the distances covered either in defense or offense. Other discriminant variables included participation in offense (touches and front court touches) and passing-related variables (passes, assists, secondary assists, assists opportunities and points created by assists). There are also unique traits from each cluster that could be used to optimize the training process. For example, due to their high playing times in game competition, players from cluster 2 are likely high conditioned players, however, they should also give the most concern for coaches when planning recovery time between games [42]. Conversely, players from cluster 4 comprised all guards or shooting guards with extremely high values from time of possession, touches per game or passing-related variables. This is key information for coaches to optimize representative task designs that enable players to perceive adequate environmental information and to subsequently act accordingly [25, 30, 43]. Finally, players from cluster 3 demonstrated less possession time and touches, despite the higher minutes of play, which suggests a predominant defensive role for these players. The defensive tasks are particularly related to player fitness variables as high-level defensive performances seem to require higher energy demands [44] and these kind of tasks are therefore particularly related to player fitness variables.

In addition, the worst performance variables in function 1 belong to players from cluster 1, as they exhibited lower playing times (12.6±5.0) distributed equally on playing position. In fact, the most unclear player positions (and missing values), in reference to players that can play in several different positions, were grouped in this cluster. Therefore, these results might be suggesting a profile of an all-round player that can be used in a game to serve multiple purposes, or a profile of a very specialized player (e.g. in shooting or rebounding). Together with workload compensation of reduced playing time, coaching staffs can modify the tasks to optimize the performance produced by these all-round players or specialists.

When adding the results from the second discriminant analysis function, clusters 4 and 6 emerge as active performers in the analyzed variables, such as time in possession, touches, passing, pull-up points and drives per game. These results confirm the guards profile (in cluster 4) identified previously, and also for players in cluster 6. In fact, there is an important requirement to adjust the tasks required of these players in order to fine-tune the environmental information necessary for information pick-up in game play [30, 45]. From the same perspective, players from cluster 3, identified as defensive-related, demonstrate less activity in these variables, consistent with their roles in the game.

## Conclusions

In summary, this study provided analysis of an NBA regular season using player-tracking variables and notation data. It was found that all-star players performed consistently better than non all-star players within 12 feet of the basket, possibly a result of optimized attention processes that are key for perceiving the required appropriate environmental information for action production. In addition, different groupings were identified based on playing performance, particularly in relation to the roles of scoring, passing, defensive and all-round duties. These findings can be used to optimize preparation for individual player groupings and, ultimately, improve game performances of the players and teams.

## References

[1] Gonzalez AM, Hoffman JR, Rogowski JP, Burgos W, Manalo E, Weise K, et al Performance changes in NBA basketball players vary in starters vs. nonstarters over a competitive season. Journal of strength and conditioning research / National Strength & Conditioning Association. 2013;27(3):611–5. 10.1519/JSC.0b013e31825dd2d9 22648143

[2] Schelling X, Calleja-Gonzalez J, Torres-Ronda L, Terrados N. Using Testosterone and Cortisol as Biomarker for Training Individualization in Elite Basketball: A 4-Year Follow-up Study. Journal of strength and conditioning research / National Strength & Conditioning Association. 2015;29(2):368–78. 10.1519/JSC.0000000000000642 25144130

[3] Gibson J. The ecological approach to visual perception Boston: Houghton Mifflin; 1979. 332 p.

[4] Savelsbergh G, Davids K, van der Kamp J, Bennett SJ. Development of Movement Coordination in Children: Applications in the Field of Ergonomics, Health Sciences and Sport: Taylor & Francis; 2013.

[5] Kauffman SA. The Origins of Order: Self Organization and Selection in Evolution: Oxford University Press; 1993.

[6] Karipidis A, Fotinakis P, Taxildaris K, Fatouros J. Factors characterizing a successful performance in basketball. J Hum Movement Stud. 2001;41(5):385–97.

[7] Malarranha J, Figueira B, Leite N, Sampaio J. Dynamic Modeling of Performance in Basketball. International Journal of Performance Analysis in Sport. 2013;13:377–86.

[8] Sampaio J, Janeira M. Statistical analyses of basketball team performance: understanding teams’ wins and losses according to a different index of ball possessions. International Journal of Performance Analysis in Sport. 2003;3(1):40–9.

[9] Kozar B, Vaughn RE, Whitfield KE, Lord RH, Dye B. Importance of Free-Throws at Various Stages of Basketball Games. Percept Motor Skill. 1994;78(1):243–8.

[10] Ibanez SJ, Sampaio J, Feu S, Lorenzo A, Gomez MA, Ortega E. Basketball game-related statistics that discriminate between teams' season-long success. European journal of sport science. 2008;8(6):369–72.

[11] Mikolajec K, Maszczyk A, Zajac T. Game Indicators Determining Sports Performance in the NBA. Journal of human kinetics. 2013;37:145–51. 10.2478/hukin-2013-0035 24146715 PMC3796832

[12] NBA.com. MVP Nash Highlights All-NBA First Team 2006 [April 7, 2015]. Available from: http://www.nba.com/news/AllNBA_060517.html.

[13] Maheswaran R, Chang Y-H, Henehan A, Danesis S. Deconstructing the Rebound with Optical Tracking Data. MIT Sloan Sports Analytics Conference 2012. 2012.

[14] Goldsberry K, Weiss E. The Dwight Effect: A New Ensemble of Interior Defense Analytics for the NBA. MIT Sloan Sports Analytics Conference 2012. 2012.

[15] Perše M, Kristan M, Kovačič S, Vučkovič G, Perš J. A trajectory-based analysis of coordinated team activity in a basketball game. Computer Vision and Image Understanding. 2009;113(5):612–21.

[16] Buchheit M, Allen A, Poon TK, Modonutti M, Gregson W, Di Salvo V. Integrating different tracking systems in football: multiple camera semi-automatic system, local position measurement and GPS technologies. J Sport Sci. 2014;32(20):1844–57. 10.1080/02640414.2014.942687 25093242

[17] Ben Abdelkrim N, El Fazaa S, El Ati J. Time-motion analysis and physiological data of elite under-19-year-old basketball players during competition. British journal of sports medicine. 2007;41(2):69–75; discussion 17138630 10.1136/bjsm.2006.032318 PMC2658931

[18] Leite NM, Leser R, Goncalves B, Calleja-Gonzalez J, Baca A, Sampaio J. Effect of defensive pressure on movement behaviour during an under-18 basketball game. International journal of sports medicine. 2014;35(9):743–8. 10.1055/s-0033-1363237 24816890

[19] Sampaio J, Gonçalves B, Rentero L, Abrantes C, Leite N. Exploring how basketball players' tactical performances can be affected by activity workload. Sci Sport. 2014.

[20] Aglioti SM, Cesari P, Romani M, Urgesi C. Action anticipation and motor resonance in elite basketball players. Nat Neurosci. 2008;11(9):1109–16. 19160510 10.1038/nn.2182

[21] Mangine GT, Hoffman JR, Wells AJ, Gonzalez AM, Rogowski JP, Townsend JR, et al Visual Tracking Speed Is Related to Basketball-Specific Measures of Performance in NBA Players. Journal of strength and conditioning research / National Strength & Conditioning Association. 2014;28(9):2406–14. 10.1519/JSC.0000000000000550 24875429

[22] Remmert H. Analysis of group-tactical offensive behavior in elite basketball on the basis of a process orientated model. Eur J Sport Sci. 2003;3(3):1–12.

[23] Duarte A, Davids K, Chow J, Passos P, Raab M. The development of decision making skill in sport: An ecological dynamics perspective In: Duarte A, Hubert R, editors. Perspectives on Cognition and Action in Sport. United States of America: Nova Science Publishers, Inc., Suffolk; 2009 p. 157–69.

[24] Pinder RA, Davids K, Renshaw I, Araujo D. Representative Learning Design and Functionality of Research and Practice in Sport. J Sport Exercise Psy. 2011;33(1):146–55. 10.1123/jsep.33.1.146 21451175

[25] Sampaio J, Janeira M, Ibanez S, Lorenzo A. Discriminant analysis of game-related statistics between basketball guards, forwards and centres in three professional leagues. European journal of sport science. 2006;6(3):173–8.

[26] O'Donoghue P. Research Methods for Sports Performance Analysis. London: Routledge; 2010. 278 p.

[27] Pedhazur E. Multiple Regression in Behavioral Research. Holt RW, editor. New York1982.

[28] Norusis M. SPSS 13.0 Guide to Data Analysis. Upper Saddle-River, N.J.: Prentice Hall, Inc.; 2004.

[29] Gold JI, Shadlen MN. The neural basis of decision making. Annu Rev Neurosci. 2007;30:535–74. 17600525 10.1146/annurev.neuro.29.051605.113038

[30] Davids K, Renshaw I, Glazier P. Movement models from sports reveal fundamental insights into coordination processes. Exerc Sport Sci Rev. 2005;33(1):36–42. 15640719

[31] Vilar L, Araújo D, Davids K, Button C. The role of ecological dynamics in analysing performance in team sports. Sports Med. 2012;42(1):1–10. 10.2165/11596520-000000000-00000 22149695

[32] Esteves PT, Araújo D, Davids K, Vilar L, Travassos B, Esteves C. Interpersonal dynamics and relative positioning to scoring target of performers in 1 vs. 1 sub-phases of team sports. Journal of sports sciences. 2012;30(12):1285–93. 10.1080/02640414.2012.707327 22852826

[33] Headrick J, Davids K, Renshaw I, Araujo D, Passos P, Fernandes O. Proximity-to-goal as a constraint on patterns of behaviour in attacker-defender dyads in team games. Journal of sports sciences. 2012;30(3):247–53. 10.1080/02640414.2011.640706 22176036

[34] Correa UC, Vilar L, Davids K, Renshaw I. Informational constraints on the emergence of passing direction in the team sport of futsal. European journal of sport science. 2014;14(2):169–76. 10.1080/17461391.2012.730063 24533523

[35] Hüttermann S, Memmert D, Simons DJ. The size and shape of the attentional “spotlight” varies with differences in sports expertise. Journal of Experimental Psychology: Applied. 2014;20(2):147–57. 10.1037/xap0000012 24708354

[36] Memmert D, Furley P. "I spy with my little eye!": breadth of attention, inattentional blindness, and tactical decision making in team sports. Journal of sport & exercise psychology. 2007;29(3):365–81. 17876972 10.1123/jsep.29.3.365

[37] Messersmith LL, Corey SM. The Distance Traversed by a Basketball Player. Research Quarterly American Physical Education Association. 1931;2(2):57–60.

[38] Weast JA, Shockley K, Riley MA. The influence of athletic experience and kinematic information on skill-relevant affordance perception. Q J Exp Psychol. 2011;64(4):689–706. 10.1080/17470218.2010.523474 21113859

[39] Davids K, Button C, Araujo D, Renshaw I, Hristovski R. Movement models from sports provide representative task constraints for studying adaptive behavior in human movement systems. Adaptive Behavior. 2006;14(1):73–95.

[40] Yarrow K, Brown P, Krakauer JW. Inside the brain of an elite athlete: the neural processes that support high achievement in sports. Nat Rev Neurosci. 2009;10(8):585–96. 10.1038/nrn2672 19571792

[41] Gomez MA, Lorenzo A, Ibanez SJ, Sampaio J. Ball possession effectiveness in men's and women's elite basketball according to situational variables in different game periods. J Sports Sci. 2013;31(14):1578–87. 10.1080/02640414.2013.792942 23679867

[42] Simenz CJ, Dugan CA, Ebben WP. Strength and conditioning practices of National Basketball Association strength and conditioning coaches. Journal of strength and conditioning research / National Strength & Conditioning Association. 2005;19(3):495–504. 10.1519/15264.1 16095396

[43] Esteves PT, de Oliveira RF, Araujo D. Posture-related affordances guide attacks in basketball. Psychol Sport Exerc. 2011;12(6):639–44.

[44] Apostolidis N, Nassis GP, Bolatoglou T, Geladas ND. Physiological and technical characteristics of elite young basketball players. J Sport Med Phys Fit. 2004;44(2):157–63. 15470313

[45] Davids K, Glazier P, Araujo D, Bartlett R. Movement systems as dynamical systems—The functional role of variability and its implications for sports medicine. Sports Med. 2003;33(4):245–60. 12688825 10.2165/00007256-200333040-00001

